# Low grade urothelial carcinoma mimicking basal cell hyperplasia and transitional metaplasia in needle prostate biopsy

**DOI:** 10.1590/S1677-5538.IBJU.2014.0512

**Published:** 2016

**Authors:** Julian Arista-Nasr, Braulio Martinez-Benitez, Leticia Bornstein-Quevedo, Elizmara Aguilar-Ayala, Claudia Natalia Aleman-Sanchez, Raul Ortiz-Bautista

**Affiliations:** 1 Departamento de Patología, Instituto Nacional de Ciencias Médicas y Nutrición, Distrito Federal, Mexico

**Keywords:** Carcinoma, Transitional Cell, Biopsy, Needle, Hyperplasia

## Abstract

**Purpose:**

The vast majority of urothelial carcinomas infiltrating the bladder are consistent with high-grade tumors that can be easily recognized as malignant in needle prostatic biopsies. In contrast, the histological changes of low-grade urothelial carcinomas in this kind of biopsy have not been studied.

**Materials and Methods:**

We describe the clinicopathologic features of two patients with low-grade bladder carcinomas infiltrating the prostate. They reported dysuria and hematuria. Both had a slight elevation of the prostate specific antigen and induration of the prostatic lobes. Needle biopsies were performed. At endoscopy bladder tumors were found in both cases.

**Results:**

Both biopsies showed nests of basophilic cells and cells with perinuclear clearing and slight atypia infiltrating acini and small prostatic ducts. The stroma exhibited extensive desmoplasia and chronic inflammation. The original diagnosis was basal cell hyperplasia and transitional metaplasia. The bladder tumors also showed low-grade urothelial carcinoma. In one case, the neoplasm infiltrated the lamina propria, and in another, the muscle layer. In both, a transurethral resection was performed for obstructive urinary symptoms. The neoplasms were positive for high molecular weight keratin (34BetaE12) and thrombomodulin. No metastases were found in either of the patients, and one of them has survived for five years.

**Conclusions:**

The diagnosis of low-grade urothelial carcinoma in prostate needle biopsies is difficult and may simulate benign prostate lesions including basal cell hyperplasia and urothelial metaplasia. It is crucial to recognize low-grade urothelial carcinoma in needle biopsies because only an early diagnosis and aggressive treatment can improve the prognosis for these patients.

## Introduction

Most studies on bladder urothelial carcinomas infiltrating the prostate have been done on samples of cystoprostatectomy. The frequency of infiltration varies between 12 and 55% depending on the series (1–5). In most cases the infiltrating urothelial tumors are high grade and are associated with a poor prognosis, even when detected in early stages (3–5). In contrast, information on urothelial cell carcinomas invading the prostate in needle biopsies is scarce. In most cases the urothelial carcinomas show nuclear pleomorphism, variably prominent nucleoli, increased mitoses, and necrosis, so they can be easily recognized as malignant (1, 2). We have recently observed two cases of low grade urothelial carcinomas of the bladder infiltrating into the prostate which showed scant atypia and were originally interpreted as basal cell hyperplasia (BCH) and urothelial cell metaplasia. It is important to recognize these malignant neoplasms in order to avoid an underdiagnosis of malignancy.

## Materials and Methods

Case 1-A 55 year old man presented one year before admission a reduction in the width and pressure of the urinary stream and a sensation of incomplete emptying of the bladder. Five months later he reported dysuria and postcoital hematuria. The prostate-specific antigen (PSA) was 5.2ng/mL and the digital rectal exam found induration of the right lobe. A needle biopsy was performed obtaining ten tissue core fragments. The original diagnosis (reviewed in three different hospitals) was nodular prostatic hyperplasia and basal cell hyperplasia. At our institution the prostate biopsy was originally interpreted as basal cell hyperplasia associated with stromal sclerosis. One month later, a transurethral resection of the prostate was made and the study found a neoplasm with a fungal aspect in the trigon measuring three cm. The neoplasm was resected and additional biopsies were obtained from the bladder mucosa. The transurethral prostate resection also showed infiltration by the neoplasm. The patient rejected cystoprostatectomy and was treated with chemotherapy and radiation. Five years later the patient is alive and asymptomatic without clinical or radiological evidence of recurrence or metastasis.

Case 2-A 66-year-old man presented a PSA level of 6.7ng/mL, obstructive urinary symptoms, dysuria, and microscopic hematuria. His physical exam was normal and the digital rectal examination revealed an enlarged prostate and induration of the left lobe. A needle biopsy was done obtaining 12 tissue core biopsies. The original diagnosis was prostatic nodular hyperplasia, basal cell hyperplasia and urothelial metaplasia. Laboratory data, chest X-ray, bone scan, and abdominal CT scan were normal. One week later, a transurethral resection of the prostate was performed, finding a bladder neoplasm with exophytic features measuring 2.5cm in the posterior wall of the bladder. The patient did not return to the hospital and his subsequent course is unknown.

## Histological Findings

The histological findings of low-grade urothelial carcinoma in needle biopsies are summarized in Table-1. The neoplasm affected two out of ten core biopsies in case 1 (Figures 1A, 1B, 2A, 2B), and one out of twelve in case 2 (Figures 3A, 3B). The morphology in both cases was similar, however in the second case the neoplasm was seen only in isolated histological fields (Figure-3A). Both needle biopsies showed chronic inflammation and extensive sclerosis of the prostatic stroma producing an irregular distribution of the ducts and acini (Figures 1A, 1B, 3A). Acini and prostatic ducts were found infiltrated by neo-plastic cells producing complete obstruction of the lumen (Figures 1A, 1B, 2A, 2B, 3A, 3B). The cells were small, basophilic and showed mild atypia or no atypia simulating basal cells (Figures 2A, 3A), while others showed perinuclear clearing, mimicking urothelial metaplasia (Figure-3B). In case 2, in addition to in situ carcinoma, small atypical nests were observed, consistent with acinar versus focal stromal infiltration (Figure-3A). Definitive diagnosis in case 1 was low-grade urothelial carcinoma in situ. Case 2 corresponded to an in situ carcinoma with probable stromal infiltration.

**Table 1:** Needle biopsies with low-grade urothelial carcinoma infiltrating the prostate. Histological findings in needle biopsy.

A) Irregular distribution of acini and ducts in the prostatic stroma.
B) Extensive stromal sclerosis associated with chronic inflammation.
C) Acini and prostatic ducts infiltrated by small, basophilic neoplastic cells with mild atypia or without atypia, sometimes with perinuclear clearing.
D) The neoplastic cells may be within the ducts (carcinoma in situ) or associated with prostatic stromal infiltration.

**Figure 1 A:**
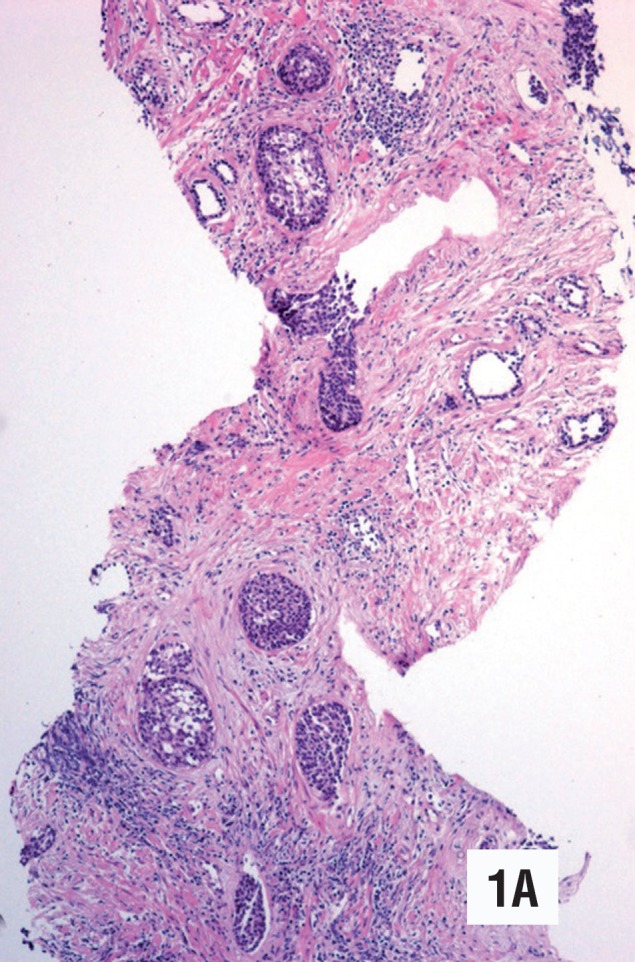
Low grade urothelial carcinoma in needle prostatic biopsy. Prostatic ducts show infiltration by malignant urothelial cells. Note the nests of basophilic and clear cells with an irregular distribution. The stroma shows sclerosis and chronic inflammation. (Hematoxylin & Eosin. Original magnification × 95).

**Figure 1 B:**
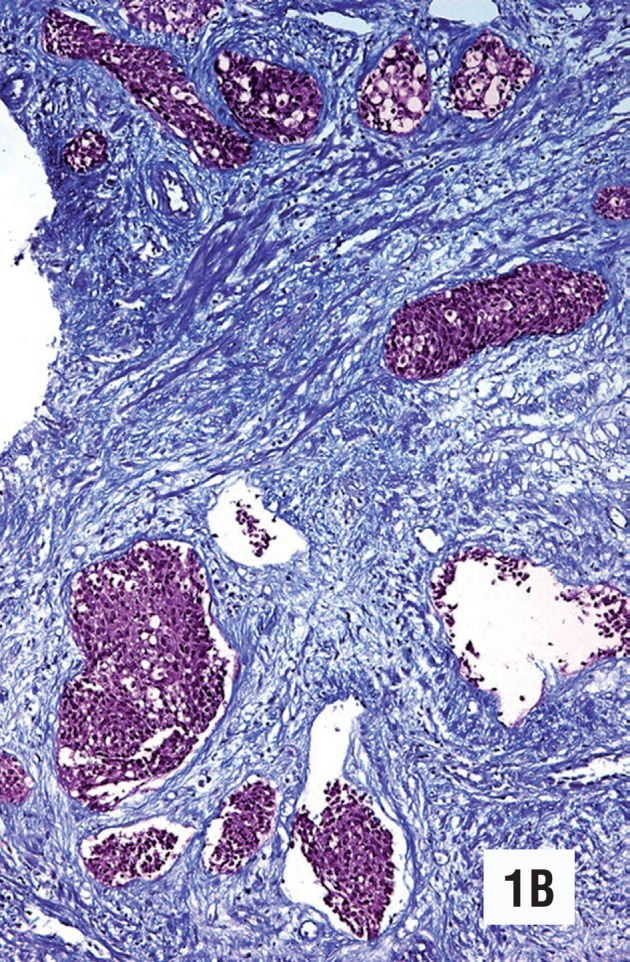
Masson stain. The stroma shows extensive desmoplasia. The tumor is limited to the prostatic ducts. (Original magnification × 110).

**Figure 2 A:**
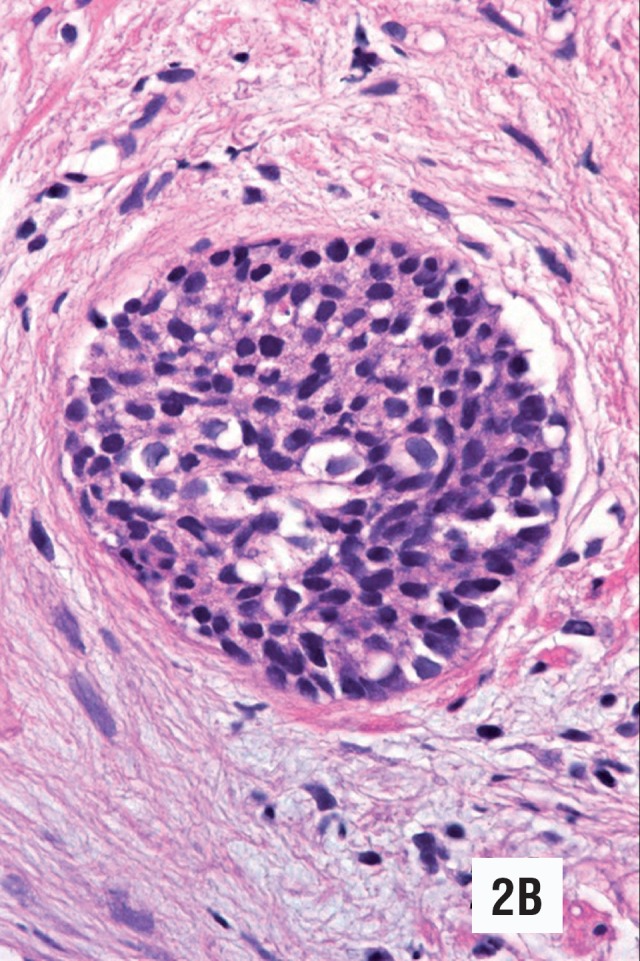
Low-grade urothelial carcinoma totally occupying prostatic duct. The cells show minimal atypia and resemble hyperplastic basal cells. (Hematoxylin & Eosin. Original magnification × 300).

**Figure 2 B:**
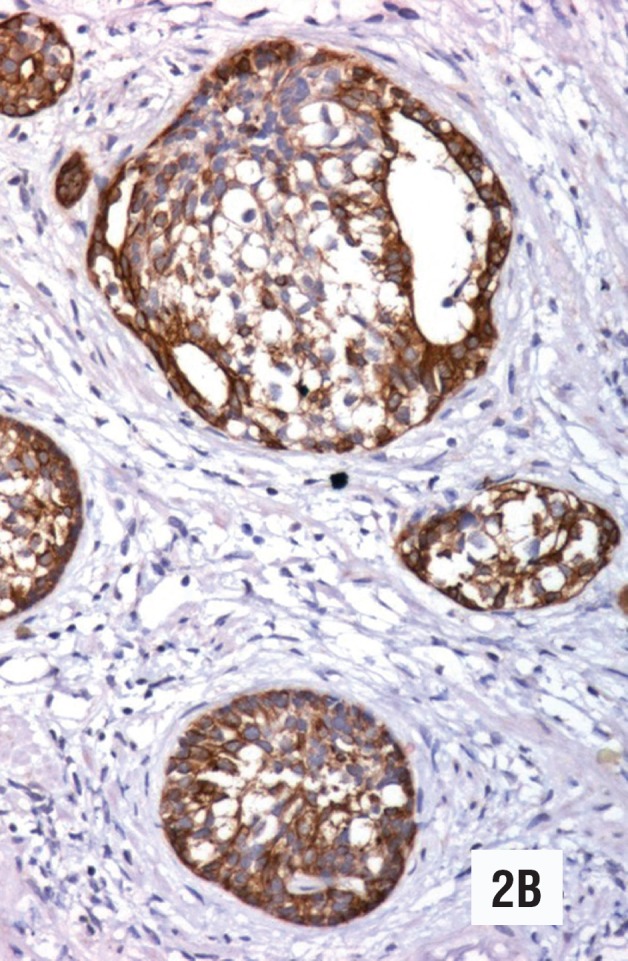
The immunohistochemical study with high molecular weight keratin (34BetaE12) is strongly positive in the neoplastic cells. (Original magnification × 270).

**figure 3 A:**
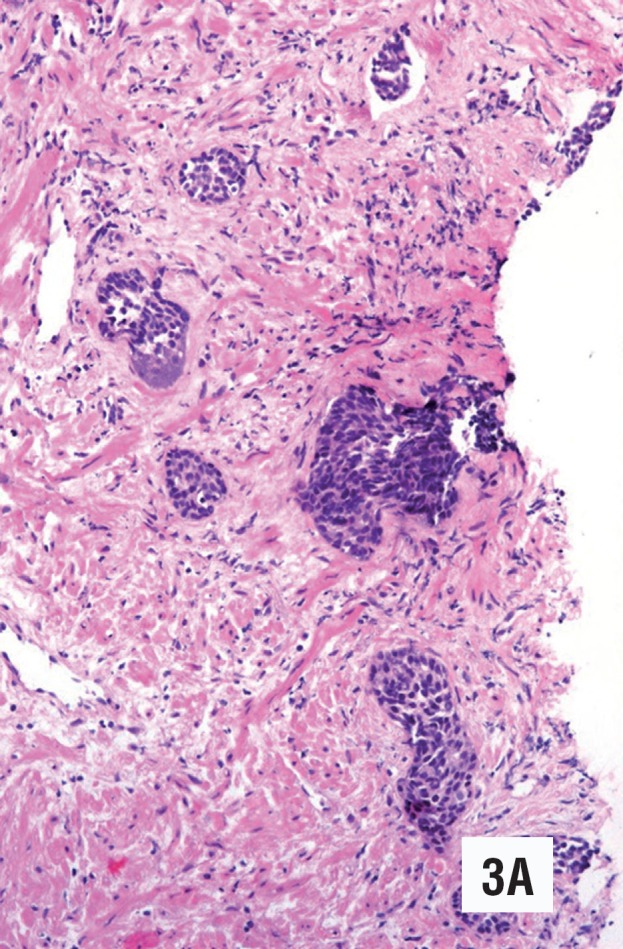
Low grade urothelial carcinoma in an isolated histological field (case 2). The smaller nests may correspond to acinar infiltration or focal stromal infiltration (Hematoxylin & Eosin. Original magnification × 110).

**Figure 3 B:**
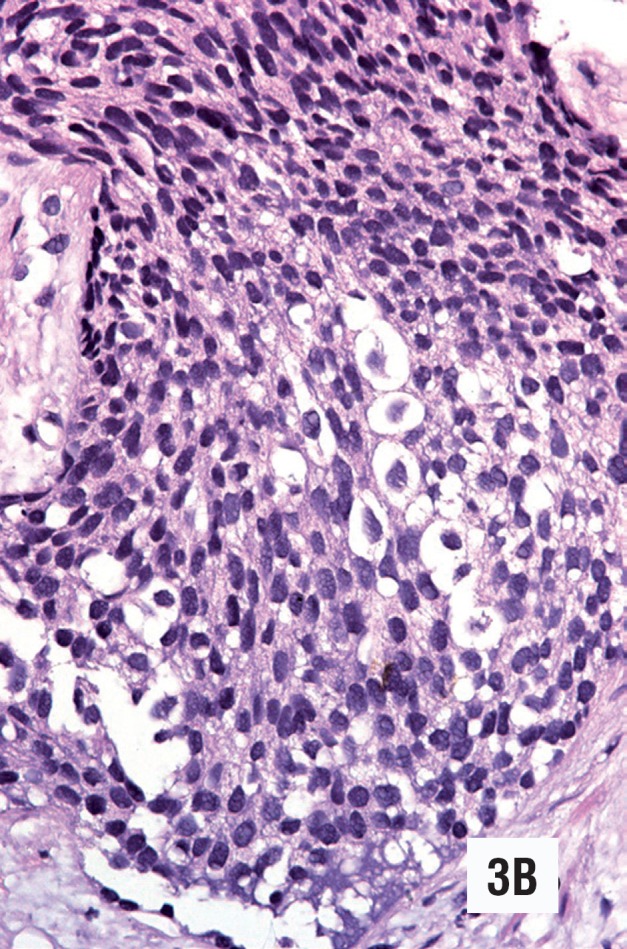
Low grade urothelial carcinoma simulating transitional cell metaplasia in needle biopsy. The cells are slightly atypical and show perinuclear clearing (Hematoxylin & Eosin. Original magnification × 300).

**Figure 4:**
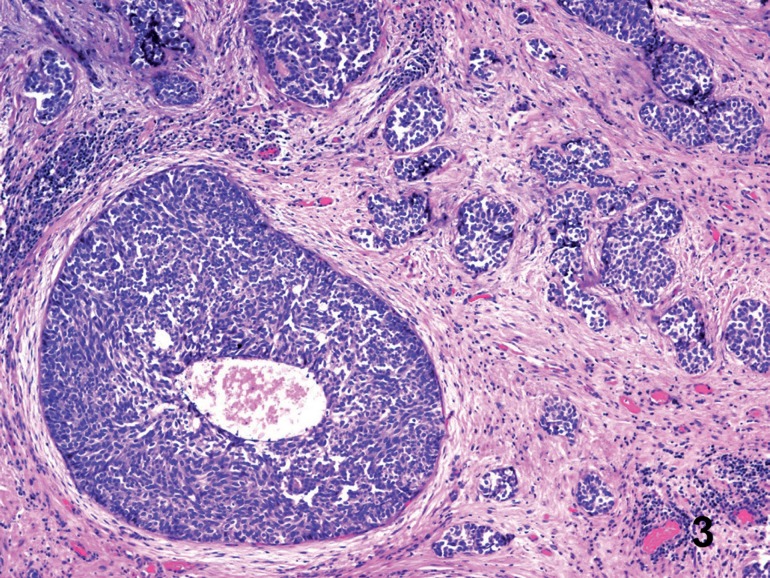
Urothelial carcinoma in transurethral resection. The neoplasm infiltrates ducts of varying diameter and comedonecrosis is observed in a mayor duct. The stroma shows desmoplasia and nests of cells with an obviously infiltrative pattern (right) (Hematoxylin & Eosin. Original magnification × 40).

Low-grade urothelial carcinoma was also seen in the bladder tumors and samples of transurethral resections. In both cases the tumor showed nests similar to those observed in the needle biopsies as well as cords of neoplastic cells with a clear infiltrative pattern that was not evident in the needle biopsies (Figure-4). Bladder tumors showed papillary growth pattern and mild atypia. In both cases there were urothelial dysplasia and in situ urothelial carcinoma. Areas with high-grade urothelial carcinoma were not found. In case one, the neoplasm infiltrated the lamina propria, and in case two the muscular layer. Immunohistochemical studies were performed on the needle biopsies and the transurethral resections. The PSA (1:50, ER-PR8, Dako) was negative in the neoplastic cells, while high molecular weight keratin (1:50, 34BetaE12, Dako) and thrombomodulin (1:25, 141C01, Thermo Scientific) were intensely positive (Figure-2B).

## Discussion

Information on the diagnosis of urothelial carcinoma infiltrating the prostate in needle biopsies is scarce. A study that included 21 patients found that most urothelial carcinomas infiltrating the prostate are high grade neoplasms, show marked atypia, and are easily recognized as malignant (1). Recently, Gordetsky and Epstein (2) reported seven cases of high-grade prostate carcinoma with pseudopapillary features mimicking urothelial carcinoma, three of them were diagnosed initially as urothelial carcinomas. All cases were diagnosed after transurethral resections. In five cases the tumor infiltrated the prostatic urethra and bladder in six.

The cases here described widen the morphological spectrum of urothelial carcinomas in needle prostatic biopsies. Both showed mild or non atypia and simulated basal cell hyperplasia and/or urothelial cell metaplasia (6–14). Although most basal cell hyperplasia are easily recognizable as benign, some cases may show a pseudoinfiltrative pattern and display apparent nucleoli (atypical basal cell hyperplasia of the prostate) (11). In addition, there are unusual variants of basal cell hyperplasia that may be difficult to diagnose (6–14). Case one was interpreted as the subtype of BCH associated with stromal sclerosis according with Devaraj and Bostwick (8). Low-grade urothelial carcinoma can also mimic urothelial metaplasia, as they are similar cytologically and both may be present within prostatic ducts in the more peripheral regions of the prostate, which might be sampled in a needle biopsy. Urothelial metaplasia generally affects isolated glands, although it can also present florid hyperplasia (6). In contrast with low-grade urothelial carcinoma, the arrangement of acini and ducts in basal cell hyperplasia and urothelial cell metaplasia is regular and the hyper-plastic nests show well defined borders (6).

Although initially the diagnosis of low grade urothelial carcinoma infiltrating the prostate was difficult to establish in needle biopsies, the immunohistochemical study and the presence of urothelial carcinoma in the bladder facilitated the interpretation in both cases. Thus, prostate biopsies showing histological changes as described herein must be studied with various antibodies, and an endoscopic examination of the bladder should also be considered in order to rule out a malignant urothelial neoplasm. The immunohistochemical study in our cases was consistent with a diagnosis of urothelial carcinoma when it turned out negative to PSA and positive to high molecular weight keratin (34BetaE12) and thrombomodulin (1, 15).

High molecular grade cytokeratin is positive in more than 90% of urothelial carcinomas (1). Other antibodies useful in the interpretation of urothelial carcinoma include CK7+, CK20+, PSA–, PAP–, and CD57– (15). According to Chuang et al. (16), PSA should be used as the first screening marker for differentiating both neoplasms. Additionally, they consider that high–molecular weight cytokeratin and P63 are more sensitive for the diagnosis of urothelial carcinoma compared with thrombomodulin and S100P. Amin et al., and the members of the ISUP Immunohistochemistry in Diagnostic Urologic Pathology Group (17) have found that on the basis of the differential diagnostic consideration, positivity for GATA3, CK20, p63, and either high–molecular weight cytokeratin or cytokeratin 5/6 are quite useful to support urothelial differentiation in the appropriate clinicopathologic context. All these antibodies are suitable in distinguishing high grade urothelial carcinomas versus poorly differentiated prostatic carcinomas because the histological and clinical distinction is frequently difficult to establish (1, 15–18).

The clinical course of patients with high-grade urothelial carcinoma diagnosed in needle biopsies is very aggressive. More than half of patients died within a few months or a few years with metastatic disease including those who showed in situ carcinoma in needle prostate biopsies (1, 15, 18). In contrast, in the two cases here studied, there was no evidence of metastasis at initial study and one of them had a five-year survival. The other patient did not return to hospital.

## Conclusions

Low-grade urothelial carcinoma infiltrating the prostate may be difficult to recognize in needle prostate biopsies. This neoplasm may be confused with benign lesions due to the absence of significant atypia. It is important to know the benign proliferations that resemble low grade urothelial carcinoma because only an early diagnosis and aggressive treatment can improve the prognosis for these patients (1, 15, 18).
